# Correlation between the MNCD classification-based staging of Parkinson’s disease and quality of life: a cross-sectional study

**DOI:** 10.1007/s00702-024-02756-4

**Published:** 2024-03-28

**Authors:** Guixiang He, Jingru Ren, Haicun Shi, Weiguo Liu, Ming Lu

**Affiliations:** 1 Department of Neurology, Affiliated Brain Hospital of Nanjing Medical University, Nanjing, People’s Republic of China; 2 The Yancheng School of Clinical Medicine of Nanjing Medical University, Yancheng Third People’s Hospital, Yancheng, People’s Republic of China; 3 Jiangsu Key Laboratory of Neurodegeneration, Department of Pharmacology, Nanjing Medical University, Nanjing, People’s Republic of China

**Keywords:** Parkinson’s disease, MNCD classification, 39-item Parkinson’s Disease Questionnaire (PDQ-39)

## Abstract

Parkinson’s disease (PD) is a highly heterogeneous neurodegenerative disorder with varying clinical subtypes. Recently, a novel classification called MNCD (Motor/Non-motor/Cognition/Dependency) has been proposed, which can also include staging based on disease severity. We aim to investigate which staging, the MNCD classification and staging or Hoehn and Yahr (H&Y) staging, exhibits a stronger correlation with the 39-item Parkinson’s Disease Questionnaire (PDQ-39). In a cross-sectional study conducted at our single center, 357 PD patients were recruited. Data encompassed scores from various assessments such as the Movement Disorder Society of the Unified Parkinson’s Disease Rating Scale (MDS-UPDRS) Parts I, II, III and IV, Montreal Cognitive Assessment (MoCA), PDQ-39, and the H&Y scale. The mean age of these patients was 66.4 ± 9.1 years old, and the majority (54.6%) were male. MNCD stages: stage 1 (*N* = 3, 0.8%), stage 2 (*N* = 62, 17.4%), stage 3 (*N* = 187, 52.4%), stage 4 (*N* = 86, 24.1%), and stage 5 (*N* = 19, 5.3%). The top 5 most frequent PD-related clinical symptoms were sleep disturbances (89.6%), fatigue (69.7%), mild cognitive impairment (68.9%), constipation (65.8%), and postural instability (65.5%). The PDQ-39 demonstrated a positive correlation with both MNCD staging and H&Y staging. Moreover, the MNCD staging exhibited a stronger correlation with PDQ-39 compared to H&Y staging. The correlation between the MNCD classification and staging with the quality of life in PD patients is more statistically significant compared to the H&Y staging.

## Introduction

Parkinson’s disease (PD) is a neurodegenerative disorder characterized by a wide range of motor and non-motor symptoms (NMS), significantly affecting patients’ health-related quality of life (Hr-QoL) and independence for activities of daily living (ADL) (Bloem and Okun 2021). PD exhibits significant clinical heterogeneity, resulting in varying symptoms and prognoses among patients, thereby posing challenges for accurate assessment of disease progression and treatment efficacy (Armstrong and Okun 2020). Therefore, various subtypes of PD have been proposed in clinical practice, including classifications based on age of onset, clinical phenotypes, neuroimaging, molecular markers, disease severity, and neuropathological alterations (Krüger et al. 2017; Lee, Park et al. 2022).

The Hoehn and Yahr (H&Y) scale, initially introduced in 1967, is extensively used to delineate the progression of PD based on its characteristic motor symptoms (Hoehn and Yahr 1967). The scale includes stages 1 through 5, with the addition of stages 1.5 and 2.5 in the 1990s to encompass the intermediate course of the disease (Jankovic et al. 1990). However, the provided information is limited to motor symptoms and does not offer a comprehensive evaluation of the patient. In recent years, the increasing recognition of the critical role of NMS in the management and diagnosis of PD has highlighted their prominent importance, as they independently influence the Hr-QoL of patients (Ren et al. 2020). Due to the significant clinical heterogeneity of PD and the substantial individual differences in symptoms and prognosis (Armstrong and Okun 2020), it becomes imperative to integrate motor symptoms and non-motor symptoms to reclassify PD and identify key symptoms across different stages of the disease. In light of this, a novel classification and staging for PD, known as MNCD, was proposed in 2021 (Santos García et al. 2021).

To date, the MNCD classification has only been utilized in a limited number of PD studies (Santos García et al. 2021; Santos-García et al. 2023a, b). However, its ability to accurately identify key symptoms and stages in PD patients remains unclear. Therefore, our objective is to classify and stage PD patients based on the MNCD classification, as well as investigate the correlation between the 39-item Parkinson’s Disease Questionnaire (PDQ-39) and both MNCD staging and H&Y staging, given that PDQ-39 is a widely employed tool for assessing quality of life in individuals with Parkinson’s disease.

## Methods

### Patients

A total of 357 idiopathic PD patients were recruited from the Department of Neurology at the Affiliated Brain Hospital of Nanjing Medical University between April 2021 to December 2022, based on the clinical diagnostic criteria established by the United Kingdom Parkinson’s Disease Society Brain Bank (Hughes, Daniel, Blankson et al. 1993).Exclusion criteria comprised atypical or secondary parkinsonism, clinically significant lesions visible on brain magnetic resonance imaging (MRI) scans, inability to complete the scale assessment, and the coexistence of comorbidity, sequelae, or any disorder that had could impede evaluation. During face-to-face interviews, neurologists collected clinical data such as gender, age, age at onset, disease duration, and education. A series of scales like the Movement Disorder Society of the Unified Parkinson’s Disease Rating Scale (MDS-UPDRS) Parts I, II, III, and IV scores (Goetz et al. 2008), Montreal Cognitive Assessment (MoCA) scores (Nasreddine, Phillips, Bédirian et al. 2005), and PDQ-39 were also assessed. This present study was approved by the Medical Ethics Committee of Affiliated Brain Hospital of Nanjing Medical University and conducted in accordance with the Declaration of Helsinki. Prior to participating in the experiment, all individuals provided written informed consent.

### Study design

The MNCD consists of four main axes: Motor Symptoms (M), Non-motor Symptoms (N), Cognition (C), and Dependency for ADL (D) (Santos García et al. 2021). Motor Symptoms (M) are assessed using MDS-UPDRS Part II, III, and IV, while Non-motor Symptoms (N) are evaluated with MDS-UPDRS Part I and PDQ-39. Cognition (C) is assessed through MoCA, in accordance with the diagnostic criteria for cognitive impairment in Parkinson’s disease (Nasreddine, Phillips, Bédirian et al. 2005; Goldman, Holden, Litvan et al. 2018). Lastly, Dependency for ADL (D) is measured using PDQ-39. It is crucial to conduct this assessment in a standardized manner by trained professionals to ensure accuracy and consistency in results.

**Fig. 1:**
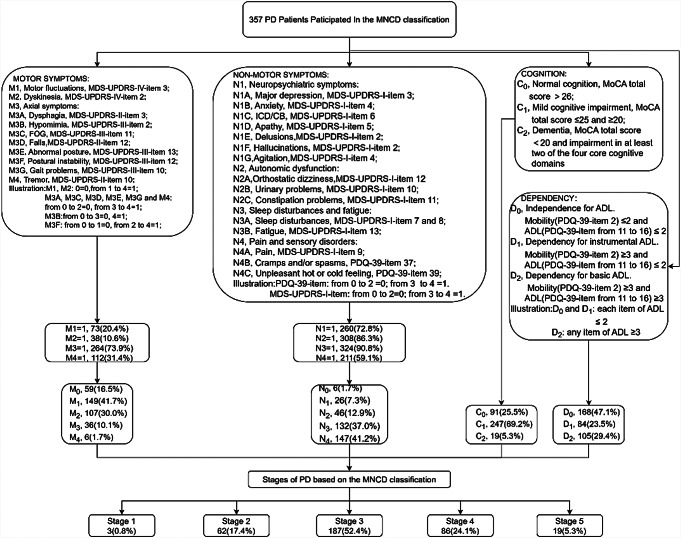
Summarizes patient enrollment process and the MNCD classification and staging. *Note.* MNCD: Motor symptoms, Nonmotor symptoms, Cognition, and Dependency for ADL; MDS-UPDRS: Movement Disorder Society-sponsored revision of the Unified Parkinson’s Disease Rating Scale; PDQ-39: 39-item Parkinson’s disease Questionnaire; MoCA: Montreal Cognitive Assessment; ADL: Activities of Daily Living; FOG: Frozen of Gait

The MNCD classification meticulously divides axes M and N into four sub-axes, noting the presence or absence of symptoms for each. If clinically relevant symptoms are detected, a sub-axis scores 1;otherwise 0. The C axis focuses on cognition with three exclusive options: normal (0), mild cognitive impairment (1), and dementia (2). The D axis measures dependency in ADL, divided into three options: independence (0), dependency in instrumental but not basic ADL (1), and dependency in basic ADL (2). Based on this classification of MNCD, we have categorized PD patients into five stages, ranging from mild symptoms (stage 1) to severe impairment (stage 5). Stage 1 is marked by no significant motor or non-motor symptoms, independence in basic ADL, and no cognitive impairment. Stage 2 includes at least one significant disabling motor or non-motor symptom, but no cognitive impairment or ADL dependency. Stage 3 is characterized by mild cognitive impairment (C = 1) and/or dependency in instrumental ADL (D = 1). Stage 4 indicates dependency in basic ADL (D = 2), while stage 5 represents dementia (C = 2) and functional dependency in basic ADL (D = 2). Figure 1 offers a comprehensive summary of the patient enrollment process, the MNCD classification, and staging.

According to Jankovic’s motor subtype classification, patients were categorized as tremor dominant (TD), indeterminate, or postural instability and gait difficulty (PIGD) (Jankovic et al. 1990). The H&Y scale, derived from the MDS-UPDRS III, is a well-established method for evaluating the severity of PD. It assesses motor symptoms on a scoring range of 1 to 5, where 1 indicates mild and 5 indicates severe (Goetz et al. 2008). In our study, we used both the MNCD staging and the H&Y staging to assess PD severity. Furthermore, we examined the correlation between these clinical staging methods and the PDQ-39 to determine which approach better reflects disease severity and quality of life among PD patients. The PDQ-39 (Peto and Jenkinson 1998) is utilized as an assessment tool for measuring Hr-QoL of patients (Den Oudsten, Van Heck, De Vries 2007). This comprehensive questionnaire measures eight domains encompassing mobility, activities of daily living, emotional well-being, stigma perception, social support, cognition function, communication ability, and bodily discomfort using a four-point scale ranging from ‘never’ to ‘always’.

### Statistical analysis

The statistical analysis was conducted using IBM SPSS software version 27.0. Continuous variables were presented as mean ± standard deviation (SD) or median (interquartile range), while categorical variables were expressed as n (%). Normality tests were performed using the Kolmogorov-Smirnov test. Descriptive data were reported in frequency and percentage. ANOVA was used for normally distributed continuous variables in multigroup comparisons, with Bonferroni correction for post hoc analysis. The Independent-Samples Kruskal-Wallis Test was engaged for multigroup comparisons of ordinal or non-normally distributed continuous variables. Chi-square tests were used to compare ratios. The Spearman rank correlation coefficient was used to depict the correlation between continuous and categorical variables, which were categorized as negligible (< 0.3), low (0.3–0.5), moderate (0.5–0.7), high (0.7–0.9), or very high (0.9-1.0) (Mukaka 2012). The permutation test was employed to compare the two Spearman rank correlation coefficients among MNCD stage, H&Y stage, and PDQ-39. Statistical significance was set at *P* < 0.05 with a 95% confidence interval (CI).

## Results

The demographic and clinical characteristics of 357 PD patients are presented in Table 1. The PD patients had an average age of 66.4 years, comprising 54.6% males (195 individuals), and exhibited a median disease duration of 7.0 years.

**Table 1:** Demographic and clinical characteristics of Parkinson’s disease patients

Demographic and Clinical Characteristics	Total n = 357
Sex (%)	
Male	195 (54.6%)
Female	162 (45.4%)
Age (years)	66.4 ± 9.1
Age at onset (years)	59.2 ± 9.8
Disease duration (years)	7.0 (4.0, 10.0)
Formal education (years)	9.0 (6.0, 12.0)
MoCA	22.0 (17.5, 25.0)
PDQ-39 total score	30.2 ± 21.1
MDS-UPDRS total score	64.3 ± 25.9
MDS-UPDRS II	13.3 ± 7.3
MDS-UPDRS III	38.1 ± 16.6
H&Y Staging	2.0 (2.0, 3.0)
Motor phenotype (%)	
TD	72 (20.2%)
PIGD	253 (70.8%)
Indeterminate	32 (9.0%)

Data are given as mean ± SD, n (%) and median (interquartile range)

MoCA: Montreal Cognitive Assessment; MDS-UPDRS: Movement Disorder Society-sponsored revision of the Unified Parkinson’s Disease Rating Scale; TD: tremor dominant; PIGD: postural instability/gait difficulty; H&Y: Hoehn and Yahr scale; PDQ-39: 39-item Parkinson's disease Questionnaire

**Fig. 2:**
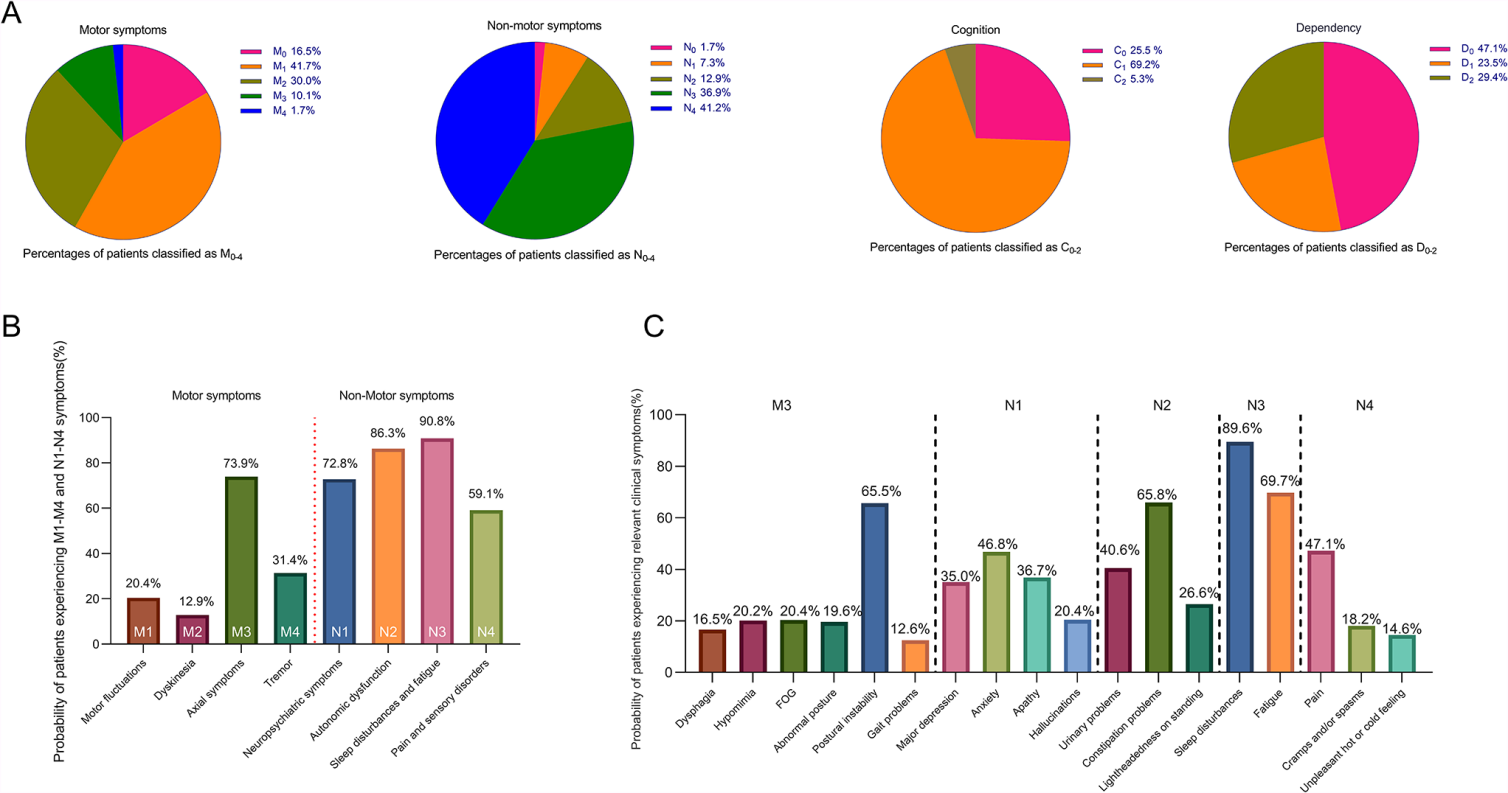
**A**) Percentages of patients classified as M_0-4_, N_0-4_, C_0-2_, and D_0-2_. M_0_, no sub-axis with symptoms; M_1_, 1 sub-axis with symptoms; M_2_, 2 sub-axes with symptoms; M_3_, 3 sub-axes with symptoms; M_4_, all sub-axes with symptoms; N_0_, no sub-axis with symptoms; N_1_, 1 sub-axis with symptoms; N_2_, 2 sub-axes with symptoms; N_3_, 3 sub-axes with symptoms; N_4_, all sub-axes with symptoms; C_0_, normal cognition; C_1_, mild cognitive impairment; C_2_, dementia; D_0_, independency for ADL; D_1_, dependency for instrumental ADL; D_2_, dependency for basic ADL. **B**) and **C**) Probability of patients experiencing relevant clinical symptoms; FOG: Frozen of Gait

Based on the MNCD classification, Fig. 2A illustrates that 16.5% (59) of patients exhibited no significant motor symptoms (M_0_), while 1.7% (6) showed no non-motor symptoms (N_0_). Only 1.7% (6) of patients presented associated motor symptoms across all sub-axes of axis 1 (M_4_). In contrast, a substantial proportion of patients, accounting for 41% (147), experienced symptoms related to all sub-axes of axis 2 (N_4_). Additionally, it is noteworthy that cognitive function remained intact in only a quarter (25.5%) of the PD patients examined in this study (C_0_); however, nearly half demonstrated independence in ADL (D_0_). According to Fig. 2B, a substantial proportion of patients (83.5%) experienced clinically significant motor symptoms, predominantly characterized by axial symptoms (73.9%). Moreover, this study also revealed that nearly all patients (98.4%) were affected by NMS, with sleep disorders and/or fatigue accounting for the highest proportion (90.8%). Furthermore, Fig. 2C highlights postural instability as the most prevalent axial symptom (65.5%). The most commonly reported NMS manifestations included sleep disorders (89.6%), fatigue complaints (69.7%), constipation problems (65.8%), pain conditions (47.1%), and anxiety disorders (46.8%).

**Fig. 3:**
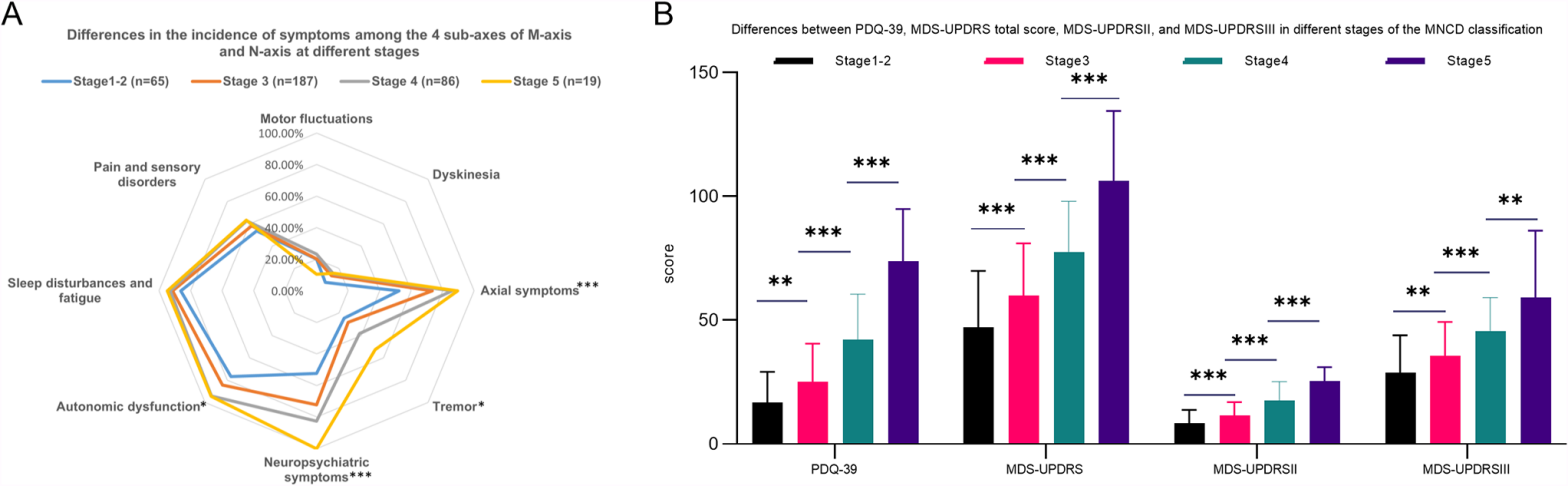
**A**) Differences in the incidence of motor and non-motor symptoms at different MNCD stages; M-axis: motor fluctuations, dyskinesia, axial symptoms, and tremor; N-axis: neuropsychiatric symptoms, autonomic dysfunction, sleep disturbances and fatigue, and pain and sensory disorders. **B**) Differences between PDQ-39, MDS-UPDRS total score, MDS-UPDRS III, and MDS-UPDRS III at different MNCD stages; MDS-UPDRS: Movement Disorder Society-sponsored revision of the Unified Parkinson’s Disease Rating Scale; PDQ-39: 39-item Parkinson’s disease Questionnaire

Table 2 presents the demographic and clinical characteristics of PD patients, categorized based on the MNCD staging. The most prevalent group was stage 3 (*n* = 187). Older age (*P* = 0.004), longer disease duration (*P* < 0.001), lower MoCA scores (*P* < 0.001), and a higher proportion of postural instability and gait difficulty with motor phenotype (PIGD) (*P* = 0.019) were associated with higher MNCD stages. Fig. 3 A illustrates significant variations in the M-axis and N-axis among different stages according to the MNCD classification and staging, encompassing sub-axes such as axial symptoms, tremor, neuropsychiatric symptoms, and autonomic dysfunction. Moreover, based on Fig. 3B, we conducted a comparative analysis of the MDS-UPDRS total score, MDS-UPDRS II, MDS-UPDRS III for motor function assessment specifically related to PD symptoms severity evaluation, as well as PDQ39 score for quality-of-life evaluation across different stages of MNCD. The findings revealed a progressive deterioration in these scores with advanced MNCD stage and significant differences between groups.

**Table 2:** Comparison of demographic and clinical characteristics among Parkinson's disease patients at different stages based on the MNCD classification

	Stage 1-2 (n = 65)	Stage 3 (n = 187)	Stage 4 (n = 86)	Stage 5 (n = 19)	p-value	post-hoc
Sex (male)	36 (55.4%)	98 (52.4%)	52 (60.5%)	9 (47.4%)	0.834	
Age (years)	63.1 ± 8.5	66.9 ± 8.7	67.0 ± 9.8	70.4 ± 8.1	**0.004**	**0.024** ^**a**^
Age at onset (years)	57.4 ± 9.7	59.7 ± 9.8	58.6 ± 10.2	62.3 ± 8.3	0.193	
Disease duration (years)	5.0 (3.0, 7.0)	7.0 (4.0, 9.0)	8.0 (6.0, 11.0)	9.0 (3.0, 11.0)	**< 0.001**	**0.029**^**a**^, **0.025**^**b**^
Formal education (years)	12.0 (7.0, 15.0)	9.0 (6.0, 12.0)	9.0 (6.0, 12.0)	9.0 (9.0, 12.0)	0.161	
Motor phenotype (%)					**0.019**	**0.037** ^**a**^
TD	22 (33.8%)	38 (20.3%)	10 (11.6%)	2 (10.5%)		
PIGD	35 (53.9%)	133 (71.1%)	69 (80.2%)	16 (84.2%)		
Indeterminate	8 (12.3%)	16 (8.6%)	7 (8.1%)	1 (5.3%)		
MoCA	26.0 (25.5, 28.0)	21.0 (17.0, 24.0)	22.0 (18.0, 24.3)	15.0 (12.0, 18.0)	**< 0.001**	**< 0.001**^**a**^, **< 0.001**^**c**^

Data are given as mean ± SD, n (%) and median (interquartile range). TD: tremor dominant; PIGD: postural instability/gait difficulty; MoCA: Montreal Cognitive Assessment. P-values calculated using ANOVA, Kruskal-Wallis H-test, or Chi-square test. Post-hoc calculated using the Bonferroni correction for multiple comparisons. ^a^ Statistically significant between stage1-2 and stage 3; ^b^ Statistically significant between stage 3 and. stage 4; ^c^ Statistically significant between stage 4 and stage 5

**Fig. 4:**
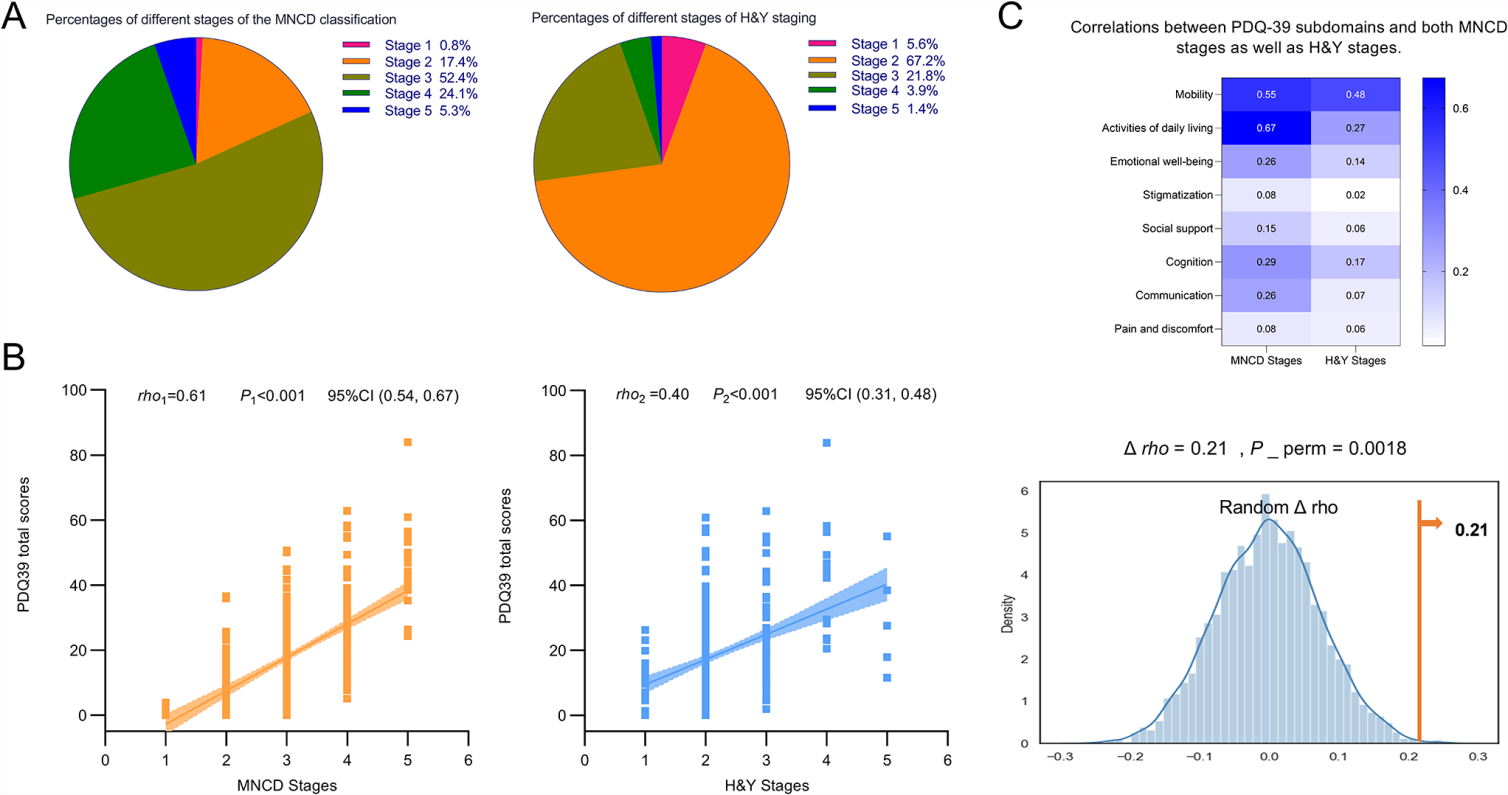
**A**) Percentages of different stages of the MNCD classification and H&Y staging. **B**) Correlations between MNCD staging and H&Y staging with respect to PDQ-39 scores; rho: Spearman’s rank correlation coefficient; P: P value; CI:Confidence Interval. **C**) Correlations between PDQ-39 subdomains and both MNCD stages as well as H&Y stages

Based on the MNCD classification and staging, Fig. 4A illustrates that approximately 52.4% (*n* = 187) of PD patients in our study were categorized as stage 3; however, according to the H&Y staging, the majority of PD patients (approximately 67.2%, *n* = 240) were classified as stage 2. Additionally, Fig. 4B demonstrates a positive correlation between MNCD staging and H&Y staging with respect to PDQ-39 scores. The permutation test revealed that compared to the H&Y staging, the MNCD staging exhibited a stronger association with PDQ-39 scores (Δ *r* = 0.21,*P*_perm =0.0018). Further analysis revealed significant correlations between MNCD staging and multiple subdomains of the PDQ-39. Specifically, these included activities of daily living (*rho* = 0.67, *P*<0.001), mobility (*rho* = 0.55, *P*<0.001), cognition (*rho* = 0.29, *P*<0.001), emotional well-being (*rho* = 0.26, *P*<0.001), and communication (*rho* = 0.26, *P*<0.001). However, H&Y staging only showed significant correlations with certain subdomains of the PDQ-39, primarily in terms of mobility (*rho* = 0.48, *P*<0.001), and activities of daily living (*rho* = 0.27, *P*<0.001) (Fig. 4C).

## Discussion

To date, the utilization of the MNCD classification has been limited to a few PD studies (Santos García et al. 2021; Santos-García et al. 2023a, b), with no existing reports comparing it to the H&Y staging. Our findings in this study demonstrate a stronger correlation between the MNCD classification and the PDQ-39 compared to the H&Y staging. This result may provide support for the utilization of MNCD classification and staging in disease management and prognosis assessment for PD patients. While various classifications have been proposed for PD in recent years (Hoehn and Yahr 1967; Goetz and Poewe 2004; Ray Chaudhuri et al. 2013; Pfeiffer 2016; Martinez-Martin et al. 2018; Aldred et al. 2020; Antonini 2021) (e.g., EOPD/LOPD based on age of onset (Schrag et al. 2000, Mehanna et al. 2022) or TD/PIGD based on motor subtype (Stebbins et al. 2013)), these do not comprehensively capture all aspects of disease severity and characteristics like the MNCD classification does.

The MNCD classification assesses PD patients based on 10 aspects, including motor fluctuations, dyskinesia, axial symptoms, tremor, neuropsychiatric symptoms, autonomic dysfunction, sleep disturbances and fatigue, pain and sensory disorders, cognitive impairment, and dependency for ADL (Santos García et al. 2021). According to the MNCD classification, our study demonstrates a higher prevalence of NMS compared to motor symptoms in PD patients. Specifically, we found that one sub-axis presentation was the most common in the M axis (41.7%), while simultaneous presence of all four sub-axes was most prevalent in the N axis (41.2%). These findings highlight the high prevalence of NMS and cognitive impairment in PD patients and suggest that staging solely based on motor symptoms is inadequate. The MNCD classification considers both motor and NMS assessments as well as evaluations of cognitive impairment and dependence on ADL (Santos García et al. 2021), making it a more comprehensive tool for assessing disease severity.

Our research findings suggest that the progression of MNCD stages is associated with several factors, including advanced age, longer disease duration, lower MoCA scores, and a higher proportion of PIGD motor phenotype. Furthermore, significant differences exist among different stages of MNCD classification in terms of axial symptoms, tremor symptoms, neuropsychiatric symptoms, and autonomic dysfunction. It is important to note that as the MDS-UPDRS total score increases along with MDS-UPDRS Part II and III scores and PDQ-39 score, the corresponding MNCD stage also increases accordingly. Stage 1 patients exhibit the highest QoL, while stage 5 patients have the poorest QoL. In conclusion, our research results indicate that the MNCD classification and staging can effectively assess both disease severity and quality of life in PD patients.

Moreover, the present study revealed that a majority of patients were categorized as stage 2 according to the H&Y staging, while most patients fell into stage 3 based on the MNCD classification and staging. Our study also demonstrated positive correlations between PDQ-39 score and both MNCD staging and H&Y staging, with the observed positive correlation between PDQ-39 scores and H&Y staging being consistent with numerous previous studies (Santos-García et al. 2023a, b; Song et al. 2014; Rahman et al. 2008). Importantly though, we observed a stronger association between the PDQ-39 total scores and the MNCD staging. These discrepancies can be attributed to two primary factors. Firstly, the H&Y scale employed in the MDS-UPDRS III defines stage 3 as mild to moderate involvement, characterized by some postural instability and assistance required during the pull test (Goetz et al. 2008). This definition differs slightly from the classical and modified H&Y scales, resulting in downgrading stages 2.5 and 3 to stage 2. Secondly, the MNCD classification encompasses four axes, with motor symptoms being just one of them (Santos García et al. 2021). The other three axes consist of NMS, cognition, and dependence for ADL. The presence of cognitive impairment or dependence for ADL can elevate the MNCD staging to at least stage 3 and even stage 5 (Santos García et al. 2021). In conclusion, the findings indicate that the MNCD staging is more consistent with the quality of daily life experienced by PD patients.

Further analysis of the PDQ-39 subdomains revealed that, in comparison to the H&Y staging, a greater number of PDQ-39 subdomains exhibited a significant correlation with MNCD staging. This suggests that MNCD classification and staging may serve as a more comprehensive and effective instrument for assessing quality of life in PD patients. However, this finding contradicts previous results reported by Galeoto et al., who observed a positive correlation between all eight subdomains of the PDQ-39 and H&Y staging (Galeoto et al. 2022). The discrepancy can be attributed to several factors: firstly, our study did not impose an age limit, whereas their study included participants aged between 50 and 90 years; secondly, their research did not encompass individuals in H&Y stage 5; thirdly, their study incorporated specific educational years and cognitive requirements; fourthly, cultural differences between Chinese and Western populations as well as translation accuracy (Luo et al. 2010).

This study represents the first attempt to compare MNCD staging with H&Y staging using PDQ-39, and it reveals a stronger correlation between MNCD staging and quality of life in PD patients. However, it also exhibits certain limitations. Firstly, due to its cross-sectional design, although it can determine the primary symptoms and disease staging of patients, it lacks the ability to monitor the progression of their condition, thereby limiting the applicability of MNCD classification. To address this issue, we plan to conduct longitudinal follow-ups in future studies to enhance the feasibility of MNCD classification. Secondly, while keeping the fundamental framework of the MNCD classification unchanged, this study made appropriate adjustments to the scales used for evaluating the required content. Although this may impose certain limitations on a comprehensive evaluation of patients’ overall condition, employing these scales in clinical practice can streamline procedures and enhance the practicality of the MNCD classification. The third point is that we only calculated the overall occurrence rate of clinically relevant symptoms associated with PD without separately evaluating each symptom occurrence rate under different stages. This limitation hinders our ability to recognize specific symptom patterns at different stages. Lastly, there was a relatively small number of patients in stage 1 and stage 5 compared to stages 2 to 4 in our sample population. Therefore, it may not fully represent all characteristics of PD and emphasizes the need for larger studies to confirm our findings.

In summary, we conducted a comprehensive assessment of PD patients using the MNCD classification and staging, and compared it with the H&Y staging by utilizing the PDQ-39. Our findings revealed a stronger correlation between the MNCD classification and staging and the quality of life in PD patients. These results suggest that the MNCD classification can serve as an effective tool for evaluating disease severity and quality of life in individuals with PD.
